# Brain Activation during Memory Encoding in Type 2 Diabetes Mellitus: A Discordant Twin Pair Study

**DOI:** 10.1155/2016/3978428

**Published:** 2016-05-29

**Authors:** Amanda G. Wood, Jian Chen, Christopher Moran, Thanh Phan, Richard Beare, Kimberley Cooper, Stacey Litras, Velandai Srikanth

**Affiliations:** ^1^Stroke and Ageing Research Group, Department of Medicine, School of Clinical Sciences, Monash University, Melbourne, VIC 3168, Australia; ^2^School of Psychology, University of Birmingham, Edgbaston B152TT, UK; ^3^Developmental Imaging, Murdoch Childrens Research Institute, Melbourne, VIC 3052, Australia; ^4^Menzies Research Institute, Hobart, TAS 7000, Australia

## Abstract

Type 2 diabetes mellitus increases the risk of dementia and neuronal dysfunction may occur years before perceptible cognitive decline. We aimed to study the impact of type 2 diabetes on brain activation during memory encoding in middle-aged people, controlling for age, sex, genes, and early-shared environment. Twenty-two twin pairs discordant for type 2 diabetes mellitus (mean age 60.9 years) without neurological disease were recruited from the Australian Twin Registry (ATR) and underwent functional magnetic resonance imaging (fMRI) during a memory encoding task, cognitive tests, and structural MRI. Type 2 diabetes was associated with significantly reduced activation in left hemisphere temporoparietal regions including angular gyrus, supramarginal gyrus, and middle temporal gyrus and significantly increased activation in bilateral posteriorly distributed regions. These findings were present in the absence of within-pair differences in standard cognitive test scores, brain volumes, or vascular lesion load. Differences in activation were more pronounced among monozygotic (MZ) pairs, with MZ individuals with diabetes also displaying greater frontal activation. These results provide evidence for preclinical memory-related neuronal dysfunction in type 2 diabetes. They support the search for modifiable later-life environmental factors or epigenetic mechanisms linking type 2 diabetes and cognitive decline.

## 1. Introduction

Type 2 diabetes mellitus is associated with an increased incidence of dementia in late life [1]. People with onset of type 2 diabetes in middle age have a greater risk of a future dementia than those with late onset disease [2] possibly because of longer exposure. Dementia, particularly Alzheimer's dementia (AD), is associated with changes in brain function that may precede overt structural brain abnormalities or cognitive deficits by several years [3]. Functional magnetic resonance imaging (fMRI) studies demonstrate alterations in the activity of distributed neural networks serving memory function in patients with early AD and its precursor state of mild cognitive impairment (MCI) and even in otherwise asymptomatic individuals at risk for AD [3]. In recent resting state fMRI studies, patients with type 2 diabetes displayed reduced functional connectivity [4] and white matter integrity [5] in the default mode network compared with controls. Cortical activation during a cognitive task may also be an important biomarker of future dementia in older people [6] and in those with type 2 diabetes [7]. Episodic encoding impairment from mesial temporal dysfunction is the hallmark early feature of cognitive decline in MCI and AD [6]. However, such an implicit encoding paradigm that provides a strong measure of mesial temporal activation [8] has not been used in the setting of type 2 diabetes and may be highly suitable to test the links between diabetes and dementia.

Genetics and early-shared environment play important roles in determining later-life brain structure [9, 10], cognition [11], and fMRI activation [12]. Therefore, accounting for confounding by genes and early-shared environment is particularly important when isolating the effects of type 2 diabetes on the earliest neural signs of impending cognitive decline, and this has not been achieved in the few previous functional imaging studies in people with type 2 diabetes. Studying cotwins who are discordant for type 2 diabetes provides powerful control for such confounders. Thus, we compared fMRI activation during implicit memory encoding, as well as cognitive function and brain structure within middle-aged twin pairs discordant for type 2 diabetes. We hypothesized that diabetes-affected individuals would have altered brain activation during a memory encoding paradigm, poorer cognitive function, and altered brain structure compared to their cotwins without diabetes.

## 2. Methods

### 2.1. Sample

The sample was derived from the Australian Twin Registry (ATR), a nation-wide volunteer agency funded by the Australian National Health and Medical Research Council (NHMRC). We invited participants (monozygotic and dizygotic) with type 2 diabetes (aged ≥50 years) and their discordant pairs without diabetes from a cohort in which type 2 diabetes was confirmed with a high degree of accuracy by a diabetologist [13]. Fasting finger-prick glucose levels was used as an additional measure of phenotype validation in our study, and fasting glucose <7.0 mmol/L was used to confirm absence of diabetes in the cotwin [14]; HbA1c levels were not available. Zygosity was established by the ATR using standard responses to questions shown to have 95% accuracy for such a purpose [15]. Exclusion criteria were a history of significant neurological disease including stroke/TIA, seizures, dementia, Parkinson's disease, and severe head trauma. This study was approved by the Monash University Human Research Ethics Committee and written informed consent was obtained.

### 2.2. MRI Acquisition

MRI scanning was performed on a single Siemens Trio Tim 3-Tesla (3T) MRI at the Murdoch Childrens Research Institute, Melbourne, Australia.* Structural MRI (sMRI) sequences*: these included high resolution T1-weighted images (0.9 mm isotropic, TR =1800 ms, TE = 2.2 ms, Flip Angle = 9°, and FOV = 230) and T2/FLAIR images (1 mm isotropic, TR = 6000 ms, TE = 405 ms, and FOV = 256);* fMRI sequences*: these included echoplanar imaging (EPI) sequence (TR = 3000 ms, TE = 40 ms, slice thickness = 3 mm no gap, FOV = 210, and N slices = 39) with whole brain coverage using an incidental memory task, based on a well-established mixed-design [16]. The person administering the task could not be completely blinded to diabetes status but was completely unaware of specific study hypotheses. The fMRI paradigm was delivered via Presentation software (Neurobehavioral Systems Inc.). The experiment comprised seven cycles of task and rest. For each cycle, three blocks of ten stimuli (words, simple line drawings, or fixation, presented for three seconds each) were shown in the centre of the screen. For blocks of words, participants were instructed to indicate via a button press whether the item was living or nonliving, to encourage encoding via a semantic route, which improves encoding [17]. Similarly, the objects depicted included animate and inanimate items and participants indicated whether these were living/nonliving via button press. There was an equal number of living and nonliving items within each block, presented in fixed random order. The resting block showed a fixation cross that varied in size. Participants indicated via button press whether the cross was large or small and were given practice outside the scanner before the session to ensure that they understood the task requirements. Each block lasted 30 seconds, giving a total session length of ten minutes and thirty seconds. The design of our experiment controlled for unwanted effects of motor response, decision-making, and visual stimulus. Participants were told that the task was designed to examine brain activity for different stimuli types and were not advised to memorize the items. They were not informed in advance of the later recognition memory test. The incidental nature of the memory encoding phase avoids directing the participant to use a particular strategy, which might account for interindividual differences in neural response. Thirty minutes after fMRI acquisition, participants were presented with a surprise recognition memory task on a laptop computer with previously presented stimuli mixed with 35 previously unseen items (“foils”). In this phase, participants were instructed to respond whether they remembered seeing the stimulus during the scanning sessions or whether it was new. Items correctly recognized in this test were assumed to reflect accurate encoding and were considered as outcomes in subsequent paired fMRI analyses.

### 2.3. Cognitive Assessment

These tests were performed after fMRI and were selected for their sensitivity to early cognitive decline in dementia. We screened general intellectual abilities with the National Adult Reading Test-Revised (NART-R) [18]; basic attention processing skills with the Mental Control and Digit Span subtests from the Wechsler Memory Scale 3 (WMS3) [19]; Memory with Hopkins Verbal Learning Test-Revised [20]; short (5 minute) recall for delayed Rey-Osterrieth Complex Figure [20]; Paired Associate Learning subtest from the Wechsler Memory Scale (WMS-1). In addition, we used the Cambridge Neuropsychological Automated Test Battery (CANTAB) [21] to measure basic and choice reaction time and visual Paired Associate Learning (VPAL), a task which is highly sensitive to the early detection of AD [22]. Paired *t*-tests were used to compare cognitive scores between cotwins.

### 2.4. Image Analyses

Preprocessing was conducted blind to age, sex, diabetes status, clinical or cognitive measurements, and zygosity.

*fMRI Analysis*. We analyzed fMRI memory blood oxygen-level dependent (BOLD) activation with FSL 4.1.4 (FMRIB Software Library, The University of Oxford) using the standard analytic pipeline. Following brain extraction with BET [23], linear registration was performed using T1 images. EPI scans of each subject were motion and slice-timing corrected, normalized to the standard Montreal Neurological Institute (MNI152) template in stereotaxic coordinate space, and smoothed using a 5 mm isotropic Gaussian kernel. After high-pass filtering (cut-off 100 s), data were convolved using Gamma function with temporal derivative and analyzed using a general linear model. We used event-related analyses to compare encoding-related activation responses for individual stimuli (drawings and words) that were correctly recognized versus activation during the baseline condition (cross-hairs) for each participant. Group-level analysis across the whole brain was first performed to characterize the pattern of activation during memory encoding in the T2DM affected and unaffected twins using *z* > 2.3 and cluster family-wise error (FWE) corrected *p* < 0.05. Where stated, we controlled for relevant health-related variables (e.g., waist circumference) by entering data in FSL during higher-level group analyses.

The study hypotheses were tested by first examining differences in activation across the whole brain, within twin pairs by paired-*t* test of the contrast images. We also used mesial temporal region-of-interest analysis to examine BOLD activation within the hippocampi based on an in-house hippocampal mask generated by the average of manual segmentation of the hippocampi in the 22 pairs, as well as hippocampal and parahippocampal masks derived from an automated anatomic labelling scheme. Standard analyses using the general linear model were first conducted in the whole sample and then repeated after stratifying for zygosity and controlling for sex (except among MZ), history of hypertension, and waist circumference by including them as variables of no interest. We additionally explored the relationship of duration of diabetes and waist circumference on regional activation among those with type 2 diabetes alone, controlling for age, sex, and hypertension. This regression analysis was performed on a voxelwise basis, across the whole brain, corrected for multiple comparisons (*Z* > 2.3, corrected (cluster) *p* = 0.05). A final, exploratory, conjunction analysis using the minimum statistic [23] was used to examine whether activation differences between discordant pairs were similar for monozygotic and dizygotic twins. 

*sMRI Analysis*. An optimized voxel-based morphometry (VBM) method was implemented in statistical parametric mapping software version 8 (SPM 8, Functional Imaging Laboratories, Institute of Neurology, London) [24]. Automated segmentation of brain from nonbrain structures was conducted followed by coregistration of the brains of all participants to the MNI152 template. VBM detects changes in the regional concentration of gray matter after correcting for global differences in brain shape. The optimized method includes recursive segmentation and spatial normalization and a modulation step to allow comparison of tissue volumes. For region-of-interest analysis, hippocampal boundaries were identified according to previously defined and validated anatomical landmarks and manually segmented by a single expert [25]. We derived whole brain gray matter, white matter, and hippocampal volumes using in-house voxel-counting algorithms. Paired comparison of global volumes was followed by paired voxelwise comparison of regional gray and white matter distribution, adopting a family-wise error (FWE) corrected *p* < 0.05. In addition, restricted paired region-of-interest (ROI) comparisons were made by applying the hippocampal mask derived from the Automated Anatomical Labelling AAL atlas [26]. 

*Cerebrovascular Lesions*. All images were reviewed by two stroke experts (Thanh Phan, Velandai Srikanth) to identify brain infarcts defined as a hypointense lesion with a hyperintense rim on FLAIR measuring greater than 3 mm. WML were manually segmented on FLAIR images by a single trained expert using established in-house methods with high test-retest reliability (intraclass correlation coefficient, ICC 0.98; 95% CI; 0.97–0.99) [27].

### 2.5. Other Measurements and Analyses

Standardized, structured questionnaires were used to record educational qualifications, self-reported medical history including hypertension, ischemic heart disease, stroke, hyperlipidemia, smoking, alcohol consumption, and prior head injury, medication use, a measure of current mood with the Geriatric Depression Scale (GDS) [28], and age of onset of diabetes. Resting arm blood pressure, waist and hip circumferences, height, and weight were obtained using standardized protocols.

## 3. Results

Sample characteristics are provided in Table 1. There were 22 discordant twin pairs with mean age of 60.9 years (SD 6.7), of whom 8 pairs were monozygotic (MZ, mean 60.6 years; SD 7.5) and 14 pairs were dizygotic (DZ, mean 61.5 years; SD 5.6). Overall duration of diabetes in affected individuals was 10.9 years (SD 9.9). In paired comparisons, those with type 2 diabetes had higher fasting blood glucose levels, waist circumference (hence weight), and self-reported frequency of hypertension and were more likely to be on cholesterol and blood pressure lowering medication than their cotwin (all *p* < 0.05). Among those with diabetes, most (20/22, 91%) were on oral antidiabetes medication and few (4/22, 18%) were on insulin. Five (23%) people reported sensory symptoms in their lower extremities, and four (18%) reported visual problems; all four cases were tested prior to scanning and confirmed as being able to view the in-scanner stimuli satisfactorily. Individuals with diabetes among the MZ group had greater mean waist circumference (114.3 cm versus 102.8 cm; *p* = 0.009) than their counterparts among the DZ group but showed similar mean fasting glucose (7.4 mmol/L versus 6.7 mmol/L; *p* = 0.28), levels of all other clinical/demographic variables, and duration of diabetes (data not shown). No participant reported other serious neurological disorders and all were independently living in the community.

### 3.1. Cognitive Function

There were no significant within-pair differences in predicted full scale intelligence quotient (NART-R FSIQ) or any other cognitive test either in the whole sample or within zygosity subgroups (Table 2). There were no significant within-pair differences in the number of correctly recognized (i.e., encoded) items administered during the fMRI scanning session.

### 3.2. fMRI BOLD Activation

**Figure 1:**
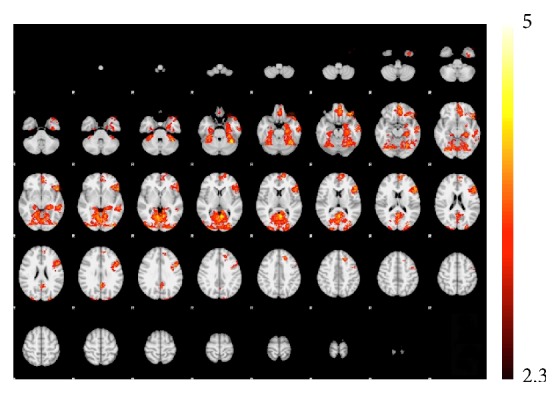
BOLD-fMRI activation during incidental memory encoding (whole-brain analysis, *Z* > 2.3, FWE corrected) in individuals without diabetes.

To confirm that the paradigm performed in the expected manner, we conducted a group analysis across the entire sample of twins without diabetes (i.e., ND). The analysis was performed only in ND because this group should demonstrate normal BOLD signal change in response to the task as well as a typical pattern of suprathreshold activation. As expected, BOLD activation for correctly encoded items during the incidental memory task was observed in the mesial temporal region as well as a distributed network of brain regions (Figure 1: *Z* > 2.3 and corrected cluster level threshold of *p* = 0.05).

#### 3.2.1. Within-Pair Comparisons among Discordant Twins

**Figure 2:**
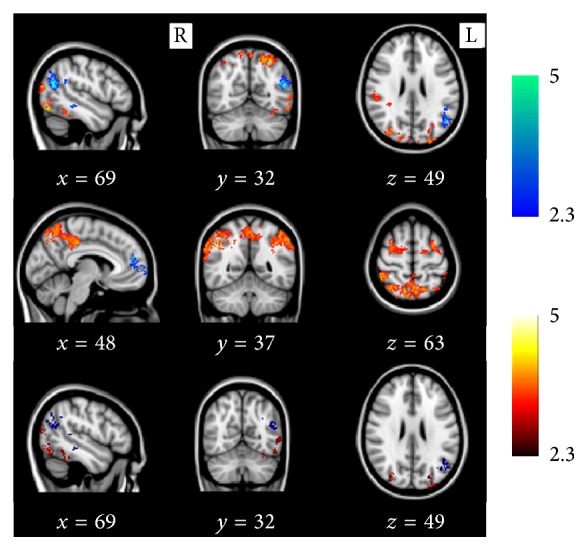
BOLD-fMRI activation differences within twin pairs discordant for type 2 diabetes. Results are displayed in all pairs (22 pairs, first row), monozygotic pairs (8 pairs, second row), and dizygotic pairs (14 pairs, last row).* Blue*: areas of decreased activation in type 2 diabetes mellitus.* Red:* areas of increased activation in type 2 diabetes mellitus. *x*, *y*, and *z* refer to Montreal Neurological Institute (MNI) voxel coordinates for the selected slices.

Reduced activation during encoding was found in type 2 diabetes in the following regions (Figure 2 (blue areas) and Table 3): (a) all pairs: left hemisphere temporoparietal structures including angular gyrus, supramarginal gyrus, and middle temporal gyrus; (b) MZ pairs: left hemisphere anterior prefrontal cortex; (c) DZ pairs: left hemisphere temporoparietal structures including middle temporal gyrus, angular gyrus, and supramarginal gyrus. Greater activation during encoding was found in type 2 diabetes in the following regions (Figure 2 (red areas) and Table 4): (a) all pairs: bilateral superior parietal lobule and precuneus, right hemisphere inferior parietal lobule, and left occipital cortex extending to fusiform and cuneus; (b) MZ pairs: bilateral widespread network spanning parietal, occipital cortices and middle frontal gyrus, and left anterior prefrontal cortex; (c) DZ pairs: bilateral precuneus, left fusiform gyrus and cuneus, and right superior and inferior parietal lobule. No within-pair differences were found in task-related BOLD activation for the subject-specific hippocampal template ROI analysis and in the ROI analysis encompassing both hippocampus and parahippocampal gyrus. In an exploratory conjunction analysis, small areas of overlap were detected between MZ and DZ comparisons for reduced activation in diabetes in the left precuneus and middle temporal gyrus and for increased activation in diabetes in bilateral superior parietal and lateral occipital cortices and the left precentral gyrus. However these clusters did not survive correction for multiple comparisons at a cluster level significance of *p* = 0.05.

#### 3.2.2. Duration of Type 2 Diabetes, Waist Circumference, and BOLD Activation

Duration of type 2 diabetes was positively correlated with left hemisphere BOLD activation in anterior prefrontal, posterior cingulate, and extrastriate cortices; right hemisphere activation in the inferior parietal, precentral gyrus, and inferior frontal operculum and bilaterally in the precuneus and angular gyrus. Waist circumference was negatively correlated with activation in left hemisphere regions including the superior and inferior frontal gyri, superior occipital gyrus, precuneus, and middle temporal gyrus.

### 3.3. Differences in Brain Volumes and Vascular Lesions

Adopting a whole brain VBM approach, there were no significant within-pair voxel differences for total gray, white, hippocampal, and WML volumes either in the whole sample or after stratifying for zygosity. Targeted region-of-interest (ROI) comparisons also showed no between-pair voxel differences in the hippocampi.

## 4. Discussion

Using a discordant pairs twin design, we found significant within-pair differences in brain BOLD-fMRI activation during memory encoding in middle-aged cotwins discordant for type 2 diabetes, in the notable absence of differences in highly sensitive measures of cognitive function or brain volumes. Type 2 diabetes was associated with reduced BOLD activation in the left temporoparietal regions, while displaying increased activation in predominantly posterior cortical networks. This increased posterior activation was particularly pronounced in MZ individuals with diabetes who also displaying greater frontal activation. Longer duration of diabetes was associated with greater activation, while greater waist circumference was associated with reduced activation. These findings provide strong support for the notion that type 2 diabetes has an adverse effect on neuronal function long before frank clinical decline is detected.

The principal strength of our study is the cotwin design supported by careful phenotyping of diabetes, the use of advanced brain imaging methods, and extensive cognitive testing. Most previous studies using fMRI in type 2 diabetes have examined activation differences in resting state [4, 29]. The use of fMRI to examine memory encoding has received little attention despite growing concern about the cognitive sequelae of diabetes in the elderly. In a recent fMRI study performed during active memory task comparing people with type 2 diabetes (*n* = 22, mean age 56 years) and controls (29, mean age 52.7 years) [7], diabetes was associated with impaired task-related deactivation of the default mode network and reduced activation of the dorsolateral prefrontal cortex. Their task differed from ours by requiring participants to actively remember an association between object and feature (colour) during the encoding phase which results in stronger prefrontal and lower mesial temporal activation [8]. In contrast, given the potential links between type 2 diabetes and Alzheimer's dementia, we focused our design by employing an incidental-encoding paradigm that reliably activates the mesial temporal lobes. We examined a slightly older sample (mean age ~60 years) and by virtue of our twin design obtained excellent control for age, genes, and markers of intellectual achievement (years of education and IQ scores on the NART-R), reflecting early-shared environment.

We propose that our data represent evidence of early alteration of functional mnemonic networks, but longitudinal data are required to confirm this interpretation. The increases in task-related BOLD activation may reflect a compensatory phenomenon to preserve function in the early stages of neuronal dysfunction or neurodegeneration in task-relevant regions. Accordingly, older patients with insulin resistance or type 2 diabetes show more diffuse regional glucose metabolism during memory encoding compared with cognitively normal older adults [30]. A recent study of 12 people with type 2 diabetes found greater left parietal and right dorsolateral prefrontal activation during high-load working memory performance compared with unrelated controls [31]. Greater levels of hyperactivation were associated with poorer disease control, providing support for the general principle that an early neural compensatory mechanism may occur in cognitively intact patients with type 2 diabetes. In contrast to our study, this study addressed higher-order executive function compensation rather than episodic memory* per se* and so the specific patterns of fMRI activation cannot be compared directly. Nevertheless, similar compensatory increases in brain activation in regions other than primary memory encoding networks are well-recognized phenomena in high-performing older adults [32] and in cognitively normal people at a higher risk of dementia such as carriers of apolipoprotein E4 (ApoE4) [33] or the presenilin 1 mutation [34]. Such increases in activation may also reflect a pathological loss of inhibition preceding deactivation [35] or purely be a physiological response to decreased activation in other regions not necessarily due to neuronal loss [36].

An interesting observation in our study was the greater BOLD activation among MZ individuals with diabetes compared with their cotwin than that observed in DZ pairs, in the presence of complete control for genetic variation and strong control for early-shared environment. Since frontal activation during a cognitive task has high heritability [12], it is conceivable that the presence of type 2 diabetes interacts with as yet undefined genotypes to influence activation in the frontal cortex. These results raise the interesting possibility that adult age environmental, lifestyle, or epigenetic factors may influence diabetes-related neuronal loss/dysfunction. Central obesity, which is closely related to adverse lifestyle, was greater among MZ individuals with diabetes in our study and is known to be associated with a greater risk of dementia [37] and brain atrophy [38], and its proinflammatory or metabolic effects on the brain may conceivably explain the stronger findings among MZ diabetics. However these results did not survive correction for multiple testing, and therefore any interpretation is speculative. The regions of increased activation are similar to those found in other studies in which compensation for early cognitive decline is mooted. Indeed, there was also evidence of reduced activation in the diabetic twin in functionally relevant areas (e.g., left middle temporal gyrus). The reduction of temporal lobe activation and concomitant increased activation in putative compensatory networks supports speculation that cognitive change experienced by older people with diabetes may be caused by neurodegeneration. An exploratory uncorrected conjunction analysis showed overlap in activation patterns in MZ and DZ twin pairs compared to their ND cotwins in these regions. This suggests that this pattern of decreased and increased activation is specifically related to the neural consequences of diabetes and therefore may represent a neural biomarker of incipient cognitive decline. We emphasise that this analysis requires replication in larger samples.

In addition to the common areas of activation or deactivation in MZ and DZ twins within the broad network of brain regions associated with memory encoding, there were a number of regions of activation that differed between MZ and DZ twins. We cannot exclude a role for glucose in the MZ-DZ differences even though blood glucose was the same between MZ and DZ diabetics, because we did not have data for HbA1c, a marker of longer-term glucose-control. There are potentially several other nongenetic factors of interest that we were unable to measure that could also explain our findings. Apart from white matter lesions, we did not have any objective measures of cerebral microvascular structure and function, aortic stiffening, physical activity, and dietary patterns. There is emerging understanding of the role of epigenetic modification of neuronal gene expression in the ageing brain and consequent impairment of neuronal function [39], and it would be of interest to study whether type 2 diabetes is associated with such changes. A recent study has demonstrated differences in histone deacetylase expression in brain between people with and without T2D, and these differences correlate with altered expression of synaptic proteins and consequent synaptic dysfunction [40].

There are limitations to our study that may be addressed in future work. The current analysis is cross-sectional, and hence causal links cannot be proven. BOLD activation also does not clearly separate the effects of changes in blood flow from actual neuronal metabolism but to counter this we excluded those with stroke, and there was no within-pair difference in the volume of white matter lesions. The effect of cerebrovascular lesions may however become more potent with advancing age and severity of type 2 diabetes and a longitudinal and/or older cohort would help answer these questions. Further research using additional and concurrent functional images methodologies (e.g., Arterial Spin Labelling, hypercapnia experiments, and neuronal metabolism using FDG-PET) may assist in further disentangling neurovascular mechanisms underlying altered cortical function in type 2 diabetes. Furthermore, cerebrovascular disease caused by diabetes may itself influence the BOLD response during cognitive tasks. For example, prescan blood glucose may influence neuronal metabolism as measured by positron emission tomography (FDG-PET). However, its impact on BOLD-fMRI activation is less clear. Hypoglycemia-related changes in activation occur only at very low glucose levels (2.5 mmol) [41], and higher levels (9.6–18.6 mmol/L) had a negligible effect on BOLD activation during a visual stimulus [42]. The physiological range of blood glucose at screening in our study (4.6–10 mmol/L) and the lack of hypoglycemic symptoms in our subjects prior to scan suggest a low likelihood for our results to be influenced by prescan glucose, but we cannot definitively exclude this. We did not have sensitive imaging measures of white matter microstructural integrity or beta-amyloid binding or genetic markers of dementia risk such as apolipoprotein epsilon 4 (ApoE4), and these have the potential to provide greater mechanistic information and potentially link AD pathology to the observed differences.

In summary, these results present evidence for dysfunctional neuronal network activity during episodic memory encoding in type 2 diabetes. The strong control for genes, age, sex, and early-shared environment in our study highlights a need to identify potentially modifiable later-life environmental/lifestyle-related or epigenetic factors that promote or modify the adverse effect of diabetes on neuronal health.

## Figures and Tables

**Table 1:** Sample characteristics (22 discordant twin pairs).

	Type 2 diabetes Mean (SD) or *n* (%)	No diabetes Mean (SD) or *n* (%)	*p* value^a^
Mean age (years)	59.5 (5.3)	59.5 (5.3)	
Male sex	12 (55%)	8 (36%)	0.38
Years of education (years)	12.9 (4.8)	12.8 (5.3)	0.88
Fasting glucose (mmol/L)	7.0 (1.5)	5.7 (0.8)	0.02
Hypertension	14 (67%)	6 (28%)	0.04
Ischemic heart disease	2 (9%)	0 (0%)	0.50
Hypercholesterolemia	12 (54%)	11 (50%)	0.98
Ever-smoking	12 (57%)	9 (41%)	0.45
Systolic blood pressure (mmHg)	151 (18.1)	142 (17.9)	0.05
Diastolic blood pressure (mmHg)	83.8 (11.6)	82.9 (8.5)	0.86
Waist (cm)	107.2 (10.6)	96.6 (16.2)	0.004
Height (cm)	164.9 (21.4)	166.4 (10.9)	0.78
Weight (kg)	93.5 (14.9)	84.1 (18.9)	0.03
Mood score (GDS)	2.7 (2.6)	2.2 (2.9)	0.45
NART-R FSIQ	107.9 (8.9)	107.8 (8.5)	0.93
HVLT delayed recall	9.0 (2.5)	9.8 (1.9)	0.25
HVLT recognition	11.5 (0.7)	11.2 (1.1)	0.25
Rey complex figure copy	34.7 (1.8)	34.7 (2.3)	0.94
Rey complex figure delayed recall	19.4 (6.1)	19.5 (6.5)	0.94
WMS3 mental control	25.5 (6.1)	25.3 (4.7)	0.88
WMS3 digit span total	18.5 (4.2)	18.6 (3.9)	0.83
PAL total errors (raw)	27.9 (30.1)	28.2 (31.5)	0.96
SRT correct latency (mean)	275.1 (62.4)	258.2 (43.5)	0.21
Total hippocampal volume (mL)	4.5 (0.5)	4.5 (0.6)	0.99
Gray matter volume (mL)	635.7 (63)	644.2 (62)	0.59
White matter volume (mL)	461.4 (59)	468.6 (52)	0.55
White matter lesion volume (mL)	4.6 (2.0)	5.1 (3.3)	0.31
MRI infarcts	0 (0)	0 (0)	
Insulin therapy	4 (18%)	0 (0)	<0.001
Oral antidiabetic agents	20 (91%)	0 (0)	<0.001
Blood pressure agents	18 (82%)	7 (32%)	<0.001
Cholesterol lowering agents	17 (77%)	5 (23%)	<0.001
Duration of diabetes (years)	10.1 (9.7)	—	

^a^ *p* for within-pair comparisons using paired *t*-test (for continuous variables) and McNemar's test (for categorical variables).

GDS: Geriatric Depression Scale; NART-R: National Adult Reading Test-Revised; HVLT: Hopkins verbal learning test; WMS3: Wechsler memory scale version 3; FSIQ: full scaled intelligence quotient; SRT: simple reaction time from the CANTAB; PAL: paired associates learning test from the CANTAB; SD: standard deviation; MRI: magnetic resonance imaging.

**Table 2:** Cognitive scores and brain volumes stratified by zygosity^a^.

Cognitive score	Monozygotic (8 pairs)		Dizygotic (14 pairs)	
	Type 2 diabetes Mean (SD)	No diabetes Mean (SD)	Type 2 diabetes Mean (SD)	No diabetes Mean (SD)
NART-R FSIQ	108.9 (8.7)	107.6 (9.9)	107.4 (9.3)	107.9 (7.9)
HVLT delayed recall	9.4 (2.1)	9.6 (1.7)	8.9 (2.7)	9.9 (2.0)
HVLT recognition	11.9 (0.4)	11.4 (1.1)	11.3 (0.8)	11.1 (1.1)
Rey complex figure copy	34.2 (2.4)	35.6 (0.7)	35.0 (1.4)	34.1 (2.7)
Rey complex figure delayed recall	19.3 (5.7)	18.5 (7.0)	19.5 (6.6)	20.1 (6.4)
WMS3 mental control	27.3 (7.2)	26.2 (5.8)	24.4 (5.4)	24.7 (4.1)
WMS3 digits total	19.6 (5.3)	19.1 (4.5)	17.8 (3.4)	18.4 (3.3)
SRT correct latency (mean)	260.9 (56.9)	273.9 (56.3)	283.1 (65.9)	249.2 (33.4)
PAL total errors (raw)	27.6 (25.2)	34.4 (41.5)	28.0 (33.6)	24.7 (25.3)
fMRI task, word errors	29.5 (16.4)	28.8 (19.8)	22.6 (15.7)	21.4 (12.2)
fMRI task, drawing errors	18.5 (14.5)	14.7 (10.9)	21.6 (14.4)	22.0 (13.7)
Total hippocampal volume (mL)	4.8 (0.4)	4.7 (0.6)	4.3 (0.5)	4.4 (0.6)
Gray matter volume (mL)	633.7 (53)	664.6 (78)	641.9 (70)	631.0 (49)
White matter volume (mL)	470.2 (48)	494.4 (56)	463.6 (63)	461.8 (45)
White matter lesion volume (mL)	5.2 (2.9)	6.2 (4.8)	4.3 (1.5)	4.5 (2.2)

NART-R: national adult reading test-revised; HVLT: Hopkins verbal learning test; WMS3: Wechsler memory scale version 3; FSIQ: full scaled intelligence quotient; SRT: simple reaction time from the CANTAB; PAL: paired associates learning test from the CANTAB; SD: standard deviation; fMRI: functional magnetic resonance imaging.

^a^None of the comparisons above reached statistical significance of *p* < 0.05.

**Table 3:** Regions (and *x*, *y*, and *z* coordinates) associated with reduced activation in type 2 diabetes.

All (22 pairs)						
Region	Hemisphere	*x*	*y*	*z*	*Z* score	*k*
Middle temporal gyrus	L	−48	−66	20	4.64	401
Angular gyrus	L	−36	−66	34	3.36	
Angular gyrus	L	−40	−68	36	3.35	
Middle temporal gyrus	L	−48	−64	32	3.29	
Inferior parietal lobule	L	−50	−46	26	3.22	
Middle temporal gyrus	L	−46	−72	30	3.08	
Middle temporal gyrus	L	−52	−36	−8	4.39	399
Middle temporal gyrus	L	−62	−46	−8	4.26	
Middle temporal gyrus	L	−62	−36	−12	3.43	
Middle temporal gyrus	L	−64	−34	−16	3.41	
Middle temporal gyrus	L	−56	−40	4	3.32	
Superior temporal gyrus	L	−62	−44	6	3.3	

MZ (8 pairs)						
Region	Hemisphere	*x*	*y*	*z*	*Z* score	*k*

Medial frontal gyrus	L	−10	58	4	4.29	270
Medial frontal gyrus	L	−6	48	18	4.27	
Superior frontal gyrus	L	−12	62	8	4.2	
Medial frontal gyrus	L	−6	48	14	4.16	
Medial frontal gyrus	L	−6	54	20	4.12	

DZ (14 pairs)						
Region	Hemisphere	*x*	*y*	*z*	*Z* score	*k*

Middle temporal gyrus	L	−60	−48	−10	3.71	421
Middle temporal gyrus	L	−52	−36	−10	3.5	
Middle temporal gyrus	L	−54	−46	8	3.42	
Middle temporal gyrus	L	−56	−36	−8	3.32	
Middle temporal gyrus	L	−60	−32	−10	3.3	
Middle temporal gyrus	L	−64	−34	−16	3.09	
Middle temporal gyrus	L	−48	−66	22	3.8	307
Angular gyrus	L	−38	−66	34	3.38	
Middle temporal gyrus	L	−38	−68	30	3.34	
Supramarginal gyrus	L	−46	−52	34	3.1	
Middle temporal gyrus	L	−42	−68	30	3.04	
Middle temporal gyrus	L	−46	−72	30	3.02	

Covariates include gender (except in MZ comparisons), waist circumference, and history of hypertension.

MZ: monozygotic; DZ: dizygotic.

*x*, *y*, and *z* refer to Montreal Neurological Institute voxel coordinates; regions are based on the Talairach Daemon; *k*: peak voxel cluster; L: left hemisphere.

All comparisons are within twin pairs and corrected for family-wise error (FWE) threshold of 0.05.

**Table 4:** Regions associated with increased activation in type 2 diabetes.

All (22 pairs)						
Region	Hemisphere	*x*	*y*	*z*	*Z* score	*k*

Precuneus	R	22	−76	50	4.85	3641
Inferior parietal lobule	R	32	−82	2	4.7	
Inferior parietal lobule	R	36	−54	48	4.59	
Superior parietal lobule	R	28	−72	50	4.49	
Superior parietal lobule	R	36	−56	58	4.36	
Cuneus	R	30	−82	6	4.34	
Fusiform gyrus	L	−46	−74	−12	4.88	1642
Cuneus	L	−2	−84	16	4.34	
Cuneus	L	−16	−90	36	4.22	
Middle occipital gyrus	L	−54	−68	−4	4.2	
Middle occipital gyrus	L	−36	−86	6	4.13	
Middle occipital gyrus	L	−46	−82	18	4.12	
Superior parietal lobule	L	−28	−64	56	4.84	540
Superior parietal lobule	L	−30	−64	50	4.32	
Superior parietal lobule	L	−24	−62	56	4.08	
Precuneus	L	−20	−60	54	4.03	
Precuneus	L	−26	−70	48	3.6	
Superior parietal lobule	L	−26	−70	52	3.6	

MZ (8 pairs)						
Region	Hemisphere	*x*	*y*	*z*	*Z* score	*k*

Precuneus	L	−4	−70	44	4.75	5640
Superior parietal lobule	R	10	−66	62	4.66	
Cingulate gyrus	R	4	−44	38	4.58	
Precuneus	R	2	−48	40	4.58	
Inferior parietal lobule	R	46	−44	60	4.56	
Superior parietal lobule	R	34	−60	60	4.53	
Angular gyrus	L	−54	−60	42	4.6	1561
Inferior parietal lobule	L	−52	−50	44	4.5	
Inferior parietal lobule	L	−38	−54	48	4.47	
Inferior parietal lobule	L	−50	−40	44	4.46	
Inferior parietal lobule	L	−52	−54	44	4.4	
Inferior parietal lobule	L	−46	−44	50	4.37	
Medial frontal gyrus	R	24	4	50	4.38	748
Medial frontal gyrus	R	32	−4	58	4.28	
Middle frontal gyrus	R	30	−4	54	4.11	
Middle frontal gyrus	R	32	6	58	3.83	
Middle frontal gyrus	L	−38	32	18	4.39	719
Middle frontal gyrus	L	−28	−2	54	4.06	
Middle frontal gyrus	L	−30	0	60	3.94	
Middle frontal gyrus	L	−38	54	−16	4.89	
Precentral gyrus	L	−24	24	34	3.78	
Precentral gyrus	L	−24	−12	54	3.74	
Precentral gyrus	L	−20	−12	54	3.74	
Superior frontal gyrus	L	−32	60	−14	4.17	
Superior frontal gyrus	L	−32	62	−18	3.87	
Middle frontal gyrus	L	−34	40	−16	3.38	328
Inferior parietal lobule	L	−60	−30	38	4.28	
Inferior parietal lobule	L	−60	−26	32	3.9	
Inferior parietal lobule	L	−66	−32	30	3.78	284
Inferior parietal lobule	L	−64	−28	30	3.7	
Inferior parietal lobule	L	−62	−36	18	3.38	
Inferior parietal lobule	L	−64	−36	24	3.23	

DZ (14 pairs)						
Region	Hemisphere	*x*	*y*	*z*	*Z* score	*k*

Middle occipital gyrus	L	−28	−92	24	4.12	857
Cuneus	L	−24	−92	28	4.07	
Cuneus	L	−16	−88	36	4.05	
Middle occipital gyrus	L	−32	−88	2	3.88	
Middle occipital gyrus	L	−24	−88	18	3.78	
Middle occipital gyrus	L	−34	−92	4	3.71	
Fusiform gyrus	L	−48	−74	−12	4.36	421
Middle occipital gyrus	L	−54	−68	−4	3.63	
Precuneus	R	22	−76	50	3.81	
Precuneus	R	26	−72	52	3.74	400
Middle occipital gyrus	R	32	−78	24	3.59	
Superior parietal lobule	R	26	−66	48	3.55	
Middle occipital gyrus	R	34	−84	4	3.52	
Precuneus	R	32	−74	40	3.34	
Superior parietal lobule	R	34	−56	58	3.65	
Inferior parietal lobule	R	36	−56	48	3.51	276
Inferior parietal lobule	R	36	−52	40	3.09	
Superior parietal lobule	R	20	−54	64	2.95	
Superior parietal lobule	R	26	−50	64	2.76	
Inferior parietal lobule	R	36	−44	46	2.57	

Covariates include gender (except in MZ comparisons), waist circumference, and history of hypertension.

MZ: monozygotic; DZ: dizygotic.

*x*, *y*, and *z* refer to Montreal Neurological Institute voxel coordinates; regions are based on the Talairach Daemon; *k*: peak voxel cluster; L: left hemisphere; R: right hemisphere.

All comparisons are within twin pairs and corrected for family-wise error (FWE) threshold of 0.05.
